# Plasma expansion and renal perfusion in critical COVID‐19 with AKI: A prospective case control study

**DOI:** 10.1111/aas.70004

**Published:** 2025-02-23

**Authors:** Tomas Luther, Per Eckerbom, Eleanor Cox, Miklos Lipcsey, Sara Bülow‐Anderberg, Michael Hultström, Jan Weis, Fredrik Palm, Susan Francis, Per Liss, Robert Frithiof

**Affiliations:** ^1^ Anaesthesiology and Intensive Care Medicine, Department of Surgical Sciences Uppsala University Uppsala Sweden; ^2^ Section of Radiology, Department of Surgical Sciences Uppsala University Uppsala Sweden; ^3^ Sir Peter Mansfield Imaging Centre, School of Physics & Astronomy University of Nottingham Nottingham UK; ^4^ NIHR Nottingham Biomedical Research Centre Nottingham University Hospitals NHS Trust and the University of Nottingham Nottingham UK; ^5^ Hedenstierna Laboratory, Department of Surgical Sciences Uppsala University Uppsala Sweden; ^6^ Integrative Physiology, Department of Medical Cell Biology Uppsala University Uppsala Sweden; ^7^ Department of Medical Physics Uppsala University Hospital Uppsala Sweden

**Keywords:** acute kidney injury, COVID‐19, fluids, magnetic resonance tomography

## Abstract

**Introduction:**

A decrease in renal perfusion during acute kidney injury (AKI) due to critical COVID‐19 has previously been demonstrated. The objective of this study was to compare the effects of plasma expansion with a standardized fluid bolus on renal perfusion in critically ill patients with AKI compared to similar patients without AKI.

**Methods:**

A case control study design was used to investigate group differences before and after a standardized intervention. ICU‐treated COVID‐19 patients without underlying kidney disease were assigned to two groups based on KDIGO Creatinine criteria for AKI. Renal perfusion was assessed by magnetic resonance imaging using phase contrast and arterial spin labeling before and directly after plasma expansion with 7.5 mL/kg Ringer's Acetate (Baxter). Arithmetic means of mean arterial pressures (MAP) recorded before and after plasma infusion were compared. Data was analyzed with a mixed model repeated measures ANOVA for all kidneys using a random effect to account for research subjects.

**Results:**

Nine patients with AKI and eight without were included in the study. The hemodynamic response to plasma expansion was similar in both groups, with increases in MAP by 9 mmHg (95% CI 0.5–18) and 15 mmHg (95% CI 5–24) in patients with and without AKI, respectively. Total renal perfusion and cortical perfusion were not significantly changed by plasma expansion in either group. There was a reduction of medullary perfusion in patients without AKI from 55 (95% CI 39–79) to 34 (95% CI 24–48) mL/min/100 g (*p* = .0027).

**Conclusion:**

Plasma expansion with a standardized fluid bolus did not increase renal perfusion in critically ill patients with COVID‐19, with or without AKI.

**Editorial Comment**

In this study, renal perfusion was measured with MRI techniques before and after plasma expansion with a crystalloid and compared between patients with and without acute kidney injury. While this resulted in increased blood pressure in both groups, there was no change in total renal perfusion or cortical perfusion, where the medullary perfusion of patients without AKI was reduced following the fluid bolus. A notable limitation is that the cases were all hemodynamically stable and therefore likely to have intact autoregulation of renal blood flow, thereby making it unclear whether or not they were hypovolemic prior to the plasma expansion. However, this is an interesting application of a technique to noninvasively measure organ‐specific blood flow in critically ill patients, and this technique could be applied to assess blood flow in other critical conditions.

## 1 INTRODUCTION

Reduced renal perfusion has been consistently reported in patients with acute kidney injury (AKI) secondary to several syndromes in critical illness, including bacterial septic shock.^1^, ^2^, ^3^

However, the notion that renal hypoperfusion is the driving mechanism in AKI development^4^ during severe infections has been challenged. This is in part due to experimental evidence of AKI development despite normal or increased renal blood flow.^5^

During the beginning of the COVID‐19 pandemic, surges of patients were admitted to intensive care units (ICUs) and treated for acute respiratory distress syndrome (ARDS). Concurrent development of AKI affected most patients and was associated with reduced chances of surviving.^6^ A close relation in time between intubation and AKI development was demonstrated^6^ and is consistent with the hypothesis that hemodynamic changes due to increased intrathoracic pressures and sedation could aggravate the loss of renal function. Whereas clinical studies of ARDS before COVID‐19 indicated the benefit of restricting intravenous fluid administration without increasing AKI incidence or severity^7^, ^8^ it was suggested that AKI in COVID‐19 was often caused by hypovolemia. Thus, meticulous care to prevent dehydration was recommended, often by treatment with intravenous fluids.^9^

Our group has previously assessed renal perfusion, oxygenation, and water content with multiparametric magnetic resonance imaging (mpMRI).^10^, ^11^ Using these methods, we demonstrated decreased renal perfusion in patients with critical COVID‐19 and AKI without a demonstrable reduction in renal oxygenation.^12^ These results are in accordance with indirect estimations of renal blood flow demonstrating that reduced renal perfusion is more common in COVID‐19 ARDS than in non‐COVID‐19 ARDS patients and that this is in turn is associated with AKI development.^13^, ^14^ Renal hypo‐perfusion may, under certain conditions, in turn, be reversed by expanding a reduced circulating plasma volume even during normotension.^15^ As renal autoregulation of blood flow is impaired during experimental studies of AKI in animals, there is a rationale to investigate whether AKI status impacts renal perfusion following plasma expansion.^16^

We hypothesized that renal perfusion can be increased by intravenous fluid treatment in ICU patients, but that the effect may be different depending on whether AKI is present or not. The objective of this study was to compare the effects of plasma expansion with a standardized intravenous fluid bolus on renal perfusion and oxygenation assessed with mpMRI in patients with AKI compared to similar patients without AKI.

## 2 METHODS

### 2.1 Study design and declarations

To compare the immediate effects on renal perfusion of plasma expansion using a standardized fluid bolus between patients with or without AKI, a case–control study design was used to investigate group differences to a standardized intervention. Interventional data were evaluated using a before–after structure to limit study participants time in the MRI scanner without adding additional arms.

The study protocol was pre‐registered and is a sub‐study of the MR‐Evaluation of Renal Function In Septic Patients (MERSEP) (ClinicalTrials ID: NCT02765191). The study was first approved by the Uppsala Regional Ethical Review Agency, Uppsala, Sweden, No. 2014/381 and 2014/381/1 (Per Erik Nistér 2017‐02‐02) with amendments No. 2020‐01996 and No. 2021‐04798 approved by the Swedish Ethical Review Authority (Maria Wetterstrand Hagström 2020‐04‐24 and 2021‐10‐26). The Declaration of Helsinki and its subsequent revisions were followed.

The study was conducted at Uppsala University Hospital, a tertiary care center in Uppsala, Sweden. The main end‐point comparisons were predefined as between‐group differences of the change in renal perfusion assessed by mpMRI to a standardized fluid bolus between two groups of patients: with AKI or no/low grade AKI (AKI group and NO AKI group). Using data from healthy volunteers, group sizes of at least *n* = 8 were calculated to have a statistical power (1‐beta coefficient) of ≥0.8 and alpha coefficient ≤0.05 for a 10% within‐group difference in renal arterial blood flow if at least one kidney was measured.^17^ All patients with at least one complete measurement of renal perfusion before and after fluid bolus from at least one kidney were included in the study.

### 2.2 Study protocol

Eligible patients admitted to the ICU were screened for inclusion. Inclusion criteria were AKI or risk of AKI development. Exclusion criteria were preexisting end‐stage renal failure or dialysis, contraindications for MRI (e.g., pace makers or certain metal implants), deterioration or instability in vital parameters to a degree where MRI was not feasible (e.g., prone positioning) and pregnancy. Informed consent was obtained from each patient or next of kin if the patient was unable to give consent. Group allocation was determined using the Kidney Disease Improving Global Outcome (KDIGO) Creatinine criteria only^18^ due to the common occurrence of oliguria without a reduction of glomerular filtration.^19^ Group allocation to the AKI group was determined as fulfillment of the KDIGO Creatinine criteria on the day of the MRI exam or within 12 h after the MRI exam. All other patients were assigned as NO AKI. Baseline Plasma (P)‐Creatinine was determined as the lowest value within a normal range during the previous 6 months up to mpMRI. Laboratory investigations were performed by the Department of Clinical Chemistry as in clinical practice, with at least daily measurement of P‐Creatinine during the ICU stay. Remaining medical data and history was collected from the electronic medical record.

Patients were transported to the MRI by ICU staff and scanned supine on a 3T MR scanner (Achieva dStream, Philips Healthcare, Best, The Netherlands) with a 16‐channel torso phased array coil together with a spine coil. Patients were ventilated with a Maquet Servo‐i MR‐Conditional ventilator (Getinge AB, Sweden) during the MRI exam with the same modality and positive end‐expiratory pressure (PEEP) as before transport and fraction of inhaled oxygen (FiO_2_), respiratory frequency and inspiratory pressures adjusted to maintain target blood oxygen saturation (SpO_2_) and minute ventilation. Sedation regime and vasoactive treatment, when present, were continued throughout the MRI exam. Saturation with pulse oximetry and invasive arterial pressure was monitored continuously and recorded manually every 5 min. An initial scan containing perfusion imaging designed to be approximately 35–40 min in length was performed first. Results from the initial scan were included in a previous publication.^12^

After the initial scan, plasma expansion was achieved by rapid infusion of 7.5 mL/kg balanced crystalloid (Ringer's Acetate, Baxter) using a manual pressure infusion bag during 5–10 min. A new assessment of renal perfusion was initiated directly after the completion of fluid bolus administration, where regional perfusion assessed with Arterial Spin Labelling (ASL) was measured first (with a duration of approximately 4–8 min) followed by total renal perfusion using phase contrast imaging (PC). Following the perfusion measurement, the left renal vein saturation was measured using T2‐Relaxation‐Under‐Spin‐Tagging (TRUST) sequence. Last, a Blood Oxygen Level Dependent (BOLD) scan was performed. The BOLD effect is sensitive to hemodilution besides deoxyhemoglobin concentration and is expected to decrease relaxation rates in blood‐rich areas, such as renal cortex, if hemodilution is present.^20^ The second exam was planned to have a duration of 15–20 min. The arithmetic mean of recorded mean arterial pressures (MAPs) during the first exam was compared with the arithmetic mean of MAPS after initiation of fluid bolus to assess hemodynamic response.

### 2.3 Perfusion and oxygenation imaging

A full description of the imaging and processing is described in the supplements of a previous publication.^12^ Regional renal perfusion was measured using a respiratory‐triggered flow‐sensitive alternating inversion recovery (FAIR) ASL sequence.^21^ An equilibrium magnetization (M0) image was collected. Post label delay (PLD) times of 400, 600, and 1800 ms were used with 5, 5, and 25 control/label image pairs collected at each PLD.^10^ Both the ASL and T1‐mapping measures collected readouts at the end of expiration. ASL data were realigned to the equilibrium *M*0 image, individual perfusion weighted difference images (control‐label) calculated, and averaged to create a perfusion‐weighted (Δ*M*) average image per PLD. T1 maps were created on a voxel‐by‐voxel basis to a standard inversion recovery equation using in‐house Matlab (The MathWorks, Inc.) code after data was inspected for motion and discarded if necessary. Tissue perfusion (*f*) maps were calculated using Δ*M* from the longest PLD of 1800 ms (with maps generated both with and without an inflow time correction from the shorter PLDs), T1 maps, and equilibrium M0 maps.^10^ T1b was assumed to be 1.55 s at 3 T,^22^ while the blood‐tissue partition coefficient *λ* was assumed to be 0.8 mL/g for kidney perfusion maps. An inflow correction time was used to assess cortex perfusion, whereas those without were used to assess medullary perfusion. Binary whole kidney masks were created by manual segmentation followed by a histogram of T1 values across both kidneys where peaks were identified to separate renal cortex and medulla. These were applied to the perfusion maps and the peak perfusion value (in mL/100 g tissue/min) for each kidney was identified with a Gaussian fit.

Phase contrast (PC)‐MRI was used to measure blood flow in each renal artery using a single slice ECG‐triggered turbo field echo. For each PC‐MRI acquisition, 20 measurements of blood flow across the cardiac cycle were collected during a single breath hold (15–20 s). PC‐MRI data of the renal arteries were analyzed using Segment software version 3.1 R8123 (http://segment.heiberg.se).^23^ A region of interest was drawn around the vessel wall, and vessel tracking was used for automatic edge detection and propagation through all phases across the cardiac cycle to calculate blood flow velocity and vessel area. Renal artery blood flow (RABF) was then calculated as the mean flux of blood flow (in mL/min) over the cardiac cycle for each renal artery. Renal artery resistance index was determined bilaterally as (*V*_systole_ − *V*_diastole_)/*V*_systole_ where *V* is the velocity of the blood flow in the renal artery measured with PC‐MRI.

TRUST data was acquired using a respiratory‐triggered FAIR‐based labeling scheme in a sagittal slice through the left renal vein. Analysis was performed in Matlab by subtracting each label from control at each effective echo time (TE) and averaging across repeats to generate the blood signal (Δ*S*(eTE)). Nine voxels with the greatest intensities within the vessel were averaged for each eTE and fit to Δ*S*(eTE) = *S*0 exp.(eTE(1/T1b − 1/T2b)) where S0 is the signal intensity of pure venous blood, T1b is the T1 of blood (assumed to be 1624 ms), to calculate the T2 of blood (T2b). A calibration curve was used to determine venous saturation (Yv) from calculated T2b and individual hematocrit level.^24^ Due to individual variation in hematocrit in response to the infusion, the R2 of blood (1/T2b), which is used in combination with hematocrit to calculate Yv, was also analyzed separately to eliminate between‐individual variations in hematocrit.

### 2.4 Statistical analysis

Measurements from the renal mpMRI were evaluated using a repeated measure ANOVA within each kidney where each individual is considered a block. First, a mixed linear model was created where fluid bolus status, AKI status, and an interaction were used as fixed effects, whereas individual and kidney were used as random effects. Model fit was assessed using visual evaluation of residuals/fitted‐ and QQ‐plots. Where heteroscedasticity was considered prevalent, logarithmic transformation was used, and the new model was similarly reevaluated. Residuals were thereafter tested for normality using Shapiro‐Wilk's method and the model accepted if normality could not be refuted. The accepted model was analyzed using ANOVA type III with Kenward–Roger's method for determination of degrees of freedom. A significant effect of either fluid bolus status or interaction, refuted the null hypothesis of no effect of fluid bolus in neither AKI nor NO AKI group and was evaluated further using pairwise comparisons for effect of plasma expansion within each group with *p*‐values adjusted using Tukey's method. *p*‐values <.05 were considered significant. A sensitivity analysis was made post hoc in which hypertension and diabetes as dichotomous variables were added to the model. Estimated marginal means were used to determine the means and confidence intervals of mpMRI measurements presented in the paper. R studio 22.07.01 using R4.1.2 was used for statistical analysis with packages lme4 (1.1–27.1), lmerTest (3.1–3), and emmeans (1.7.0) using functions lmer, anova, and emmeans. Graphs were created using the ggplot2 (3.3.5) package. Continuous data is presented as mean (95% confidence interval) in the text if not stated otherwise.

## 3 RESULTS

Seventeen patients were enrolled between June 4, 2020 and May 10, 2021 and included in the analysis. At least one measurement of P‐Creatinine within the normal range during the current hospitalization prior to the MRI examination was present in all patients. Nine were allocated to the AKI group and eight to the NO AKI group. There were 67 valid measurements of total renal blood flow and cortical perfusion and 66 measurements of medullary perfusion. Patients had a mean age of 65 (59–71) years, of which 87% (14/17) were male. All patients had ARDS at the time of the scan and of at least moderate severity at some time during their ICU stay. All but one patient were treated with invasive ventilation during the scan, with a duration of 3.1 (2.3–3.9) days. Patients were generally circulatory stable with a mean arterial pressure (MAP) of 85 (79–91) mmHg and central venous saturation of 72% (68–76). Ten patients were treated with noradrenaline using doses of 0.04 (0.02–0.06) mcg/kg/min. Urine output the hour before the exam was 1.1 (0.8–1.5) mL/kg/h. Group‐wise characteristics are presented in Tables 1 and 2.

**TABLE 1:** In a prospective case–control study investigating the effect on renal perfusions by plasma expansion by standardized balanced crystalloid bolus of 7.5 mL/kg in patients with critical COVID‐19 and ARDS divided into two groups depending on AKI status.

	AKI	NO AKI
*N*	9	8
Age, years	66 (8)	64 (15)
Female, *n* (%)	2 (22%)	1 (13%)
Height (cm)	171 (11)	178 (9)
Body weight (kg)	94 (22)	93 (20)
Body mass index (kg/m^2^)	32 (8)	29 (6)
Hypertension, *n* (%)	6 (67%)	4 (50%)
Diabetes, *n* (%)	4 (44%)	2 (25%)
ARB/ACEi treatment, *n* (%)	6 (67%)	5 (63%)
Plasma‐Creatinine baseline (μmol/L)	73 (13)	71 (16)
IHD and/or CHF, *n* (%)	2 (22%)	2 (22%)
Dexametasone‐treatment for COVID‐19, *n* (%)	8 (89%)	7 (88%)
Simplified Acute Physiology Score 3	53 (6)	54 (7)
Vasoactive treatment at any time, *n* (%)	9 (100%)	7 (88%)
Severe ARDS any time in ICU, *n* (%)	7 (78%)	5 (63%)
CRRT at any time in ICU, *n* (%)	2 (22%)	0 (0%)
Maximum AKI‐stage during hospital stay	2.2 (0.8)	0.5 (0.5)
Alive after 90 days, *n* (%)	7 (77%)	5 (63%)

*Note*: Baseline characteristics and clinical outcomes are presented by group as mean (standard deviation) or number (percentage).

Abbreviations: ACEi, Angiotensine Converting Enzyme inhibitor; AKI, acute kidney injury; ARB, Angiotensin II receptor blocking drug; ARDS, acute respiratory distress syndrome; CHD, congestive heart failure; CRRT, continuous renal replacement therapy; IHD, ischemic heart disease.

**TABLE 2:** In a prospective case–control study investigating the effect on renal perfusion by plasma expansion by standardized balanced crystalloid bolus of 7.5 mL/kg in patients with critical COVID‐19 and ARDS divided into two groups depending on AKI status.

	AKI	NO AKI
*N*	9	8
Invasive mechanical ventilation, *n* (%)	9 (100%)	7 (88%)
Days in ventilator at exam	3.4 (1.7)	2.7 (1.1)
SOFA score at exam day	7.8 (1.6)	5 (1.4)
P‐Creatinine at exam (μmol/L)	144 (104)	73 (19)
eGFR (mL/min/1.73 m^2^)	50 (17)	79 (14)
% Creatinine rise from baseline to exam	95 (113)	4 (13)
Arterial oxygen saturation (%)	95% (2)	96% (2)
Sinus rhythm	9 (100%)	7 (88%)
B‐Hemoglobin at exam (g/dL)	11.8 (1.4)	12.4 (1.2)
ScVO_2_ at exam (%)	74 (6)	70 (7)
Vasoactive drug at exam	7 (78%)	4 (50%)
Noradrenaline dose at exam (μg/kg/min)	0.05 (0.03)	0.05 (0.04)
Lactate at exam (mmol/L)	1.7 (0.8)	1.5 (0.3)
PEEP during exam, cmH_2_O	15 (4)	12 (3)
Arterial pO_2_ at exam (kPa)	10.4 (1.2)	10.7 (1.2)
Arterial pCO_2_ at exam (kPa)	6.1 (0.77)	5.6 (0.57)
Arterial pH at exam	7.38 (0.05)	7.42 (0.04)
PO_2_/FiO_2_‐ratio at exam (kPa)	22.5 (6.2)	27.4 (5.3)
Days with AKI at exam	0.9 (1.3)	
Net fluid intake at exam day (mL)	300 (522)	71 (666)
Body weight change at exam since ICU admission (%)	−1 (2)	0 (3)
nt‐ProBNP at exam (ng/L)	334 (369)	571 (521)
Latest hourly urine output at exam (mL/kg)	1.03 (0.78)	1.25 (0.63)
Furosemide within 3 h of exam, *n* (%)	22 (2)	13 (1)

*Note*: Status at exam is presented below by group as mean (standard deviation) or number (percentage).

Abbreviations: AKI, acute kidney injury; ARDS, acute respiratory distress syndrome; CRP, C‐reactive protein; eGFR, estimated glomerular filtration rate; nt‐ProBNP, n‐terminal brain natriuretic peptide; PEEP, positive end expiratory pressure; ScVO_2_, central venous saturation; SOFA, sequential organ failure assessment.

### 3.1 Hemodynamic response

Plasma expansion by the standardized fluid bolus generated an increase in mean arterial pressure and in a similar manner in both groups (Table 2). Bolus sizes administered were 712 (593–831) mL and 687 (545–828) mL in the AKI and NO AKI groups, respectively. In the group with AKI, MAP increased by 9 mmHg (0.5–18) and in the group without AKI, MAP increased by 15 mmHg (5–24). BOLD sequence during the last part of the scan demonstrated a significant reduction of cortical relaxation times in both AKI and NO AKI, consistent with hemodilution due to plasma expansion still being present after previous measurements (Table 2).

### 3.2 Renal perfusion and oxygenation

**FIGURE 1:**
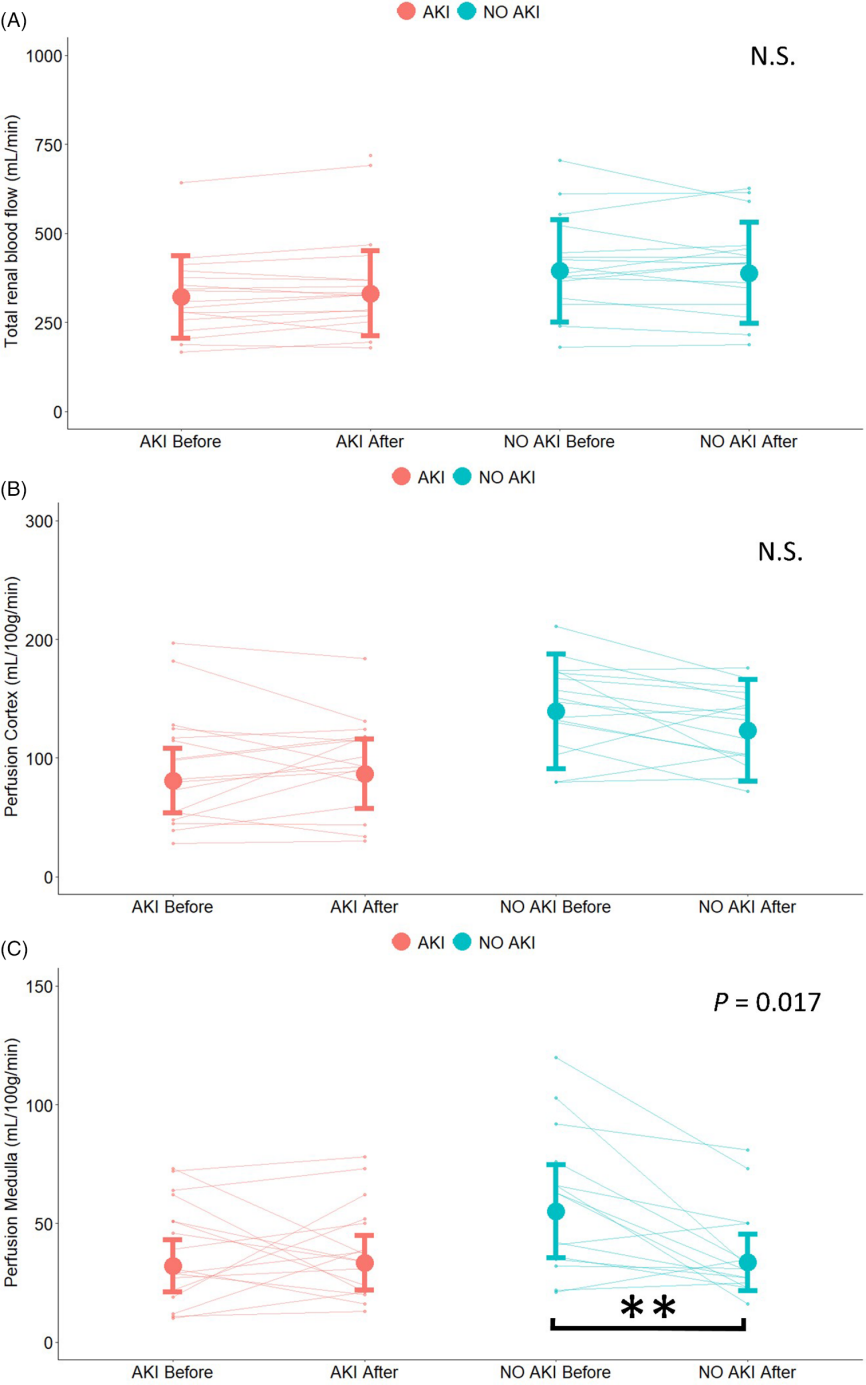
A prospective case control study investigating the effect on renal perfusion by plasma expansion using a standardized balanced crystalloid bolus of 7.5 mL/kg in patients with critical COVID‐19 and ARDS divided into two groups depending on AKI status. Main results are presented below for each measurement of perfusion using mpMRI with phase contrast estimation in (A), ASL Cortex in (B), and ASL medulla presented in (C). Individual measurements are presented as dots colorized by AKI status. Estimated marginal means and confidence intervals are presented as an overlaying bar plot. *p*‐Values in ANOVA for the interaction effect between group and plasma expansion are presented in the upper right corner of each graph. N.S. denotes non‐significant. ** denotes *p* < .01.

There were no significant effects on total renal or cortical perfusion in neither of the groups (Figure 1A,B) whereas a reduction in medullary perfusion was seen after plasma expansion in the group without AKI (Figure 1C). Total renal blood flows were 321 mL/min/kidney (224–460) before plasma expansion and 331 mL/min/kidney (231–475) after in the AKI group (N.S.). In the NO AKI group, total renal blood flows were 395 mL/min/kidney (274–568) and 389 mL/min/kidney (270–560) before and after fluid infusion (N.S.). Cortical blood flows were 81 mL/min/100 g (CI 58–113) before infusion and 87 mL/min/100 g (62–121) after (N.S.) and in the patients without AKI, cortical perfusions were 139 mL/min/100 g (98–197) before infusion and 123 mL/min/kidney (87–174) after (N.S.). Renal medullary perfusions were reduced after plasma expansion in the NO AKI group, from 55 mL/min/100 g (39–79) to 34 mL/min/100 g (24–48) (*p* = .0027) but unchanged in the AKI group, 32 mL/min/100 g (23–45) before and 33 mL/min/100 g (24–47) after (N.S.). In a sensitivity analysis made post hoc, in which hypertension and diabetes were added to the model, the results were not significantly different (Appendix S1).

Renal venous R2 was similar before in both groups and did not decrease by fluid administration; however, there were missing data from four patients (of which three are missing in AKI group) due to poor image quality (Table 3). Renal resistive index was not influenced by plasma expansion in either of the groups (Table 3).

**TABLE 3:** A prospective case control study investigating the effect on renal perfusions by plasma expansion by standardized balanced crystalloid bolus of 7.5 mL/kg in patients with critical COVID‐19 and ARDS divided into two groups depending on AKI status.

Group	AKI, *N* = 9		NO AKI, *N* = 8	
Plasma expansion status	Before	After	Before	After
Hemodynamic parameter				
MAP^††^	81 (73–88)	90 (82–98)	90 (82–98)	104 (96–113)
Renal measurements				
Resistive index	0.87 (0.78–0.97)	0.89 (0.79–0.98)	0.79 (0.70–0.89)	0.80 (0.71–0.89)
BOLD cortex^†††^	16.9 (15.7–18.0)	16.3 (15.2–17.4)***	17.4 (16.2–18.8)	16.9 (15.7–18.3)***
TRUST valid exams (% valid)	*N* = 6 (67%)	*N* = 6 (67%)	*N* = 7 (88%)	*N* = 7 (88%)
TRUST Yv	71.5 (62.2–80.8)	70.7 (61.4–80.0)	76.3 (67.7–84.9)	75.0 (66.4–83.6)
TRUST R2	10.0 (7.40–12.7)	10.6 (7.99–13.2)	10.0 (7.59–12.5)	10.4 (7.98–12.8)

*Note*: Renal perfusions and oxygenation were measured using multiparametric MRI including phase contrast, ASL, BOLD, and TRUST in which TRUST Yv is the calculated renal venous saturation derived from TRUST R2 and hematocrit. Hemodynamic parameter arithmetic mean of mean arterial pressures (MAP) before plasma expansion is presented compared with mean MAP after. Additional results are presented by group and in relation to plasma expansion status below. Data are presented as estimated marginal means with 95% confidence intervals derived from the mixed linear model used. Interaction effects were modeled without any significant effect in all analyses presented in this table. *, **, *** indicates *p*‐value <.05, 0.01, 0.001 in pairwise comparison within group after Tukey's adjustment. †, ††, ††† indicates *p*‐value <.05, .01, .001 of before‐after effect of plasma expansion in ANOVA.

## 4 DISCUSSION

In the present study, we have investigated the effects of acute plasma expansion on renal perfusion in critically ill patients with critical COVID‐19 and ARDS, and if the effects differ depending on AKI status. Our main findings are that renal perfusion and RABF did not increase after plasma expansion by a standardized balanced crystalloid bolus of 7.5 mL/kg, neither when AKI was present nor when it was not, despite generating significant increases in MAP. Also, renal medullary perfusion was lower after plasma expansion in the NO AKI group.

Neither measurements with phase contrast nor ASL could demonstrate an increase in renal perfusion in any of the groups consisting of normotensive critically ill patients. Plasma expansion by crystalloid fluid infusion is often transient with prolonged half‐lives during hypovolemia or hypotension.^25^ We assessed the renal perfusion shortly after completion of the fluid bolus when the plasma expansion likely still is present to attempt to circumvent this limitation. Further, the effects of plasma expansion were still detectable using the BOLD sequence at the end of the exam. BOLD is sensitive to deoxyhemoglobin besides hydration, but in the context of plasma expansion without changes in perfusion, present hemodilution is a probable explanation.^20^ A previous randomized study comparing plasma expansion by either crystalloid or colloid fluid bolus in ICU patients after cardiac surgery demonstrated only a transient increase in RBF attenuated completely after 60 min.^26^ In that study, larger fluid boluses were used: 20 mL/kg of crystalloid and 10 mL/kg of colloid. Larger bolus sizes than used in our study are not commonly used in clinical practice^27^ in the ICU. The bolus size chosen in the present study thus emulates the plasma expansion reasonably achievable at the bedside during similar clinical conditions. Still, the smaller volumes used in the present study resulted in a similar difference in MAP than reported in the post‐operative patients. This is likely due to administration time being shorter in our study, which was intended to maximize the plasma volume expansion while limiting increases in fluid balance. Both phase contrast and ASL are valid non‐invasive measures of renal perfusion.^10^ Using ASL, parts of our group have previously demonstrated a reduction in cortical perfusion in healthy volunteers by plasma expansion using 2 L of 0.9% saline compared with similar amounts of balanced crystalloid,^28^ a finding described well in experimental studies.^29^ Thus, when both phase contrast and cortical ASL cannot detect any significant change in renal perfusion, lack of effect is likely the cause.

In our study, renal medullary perfusion was reduced by plasma expansion in the NO AKI group but not in the AKI group, which was not an expected finding. We have previously used ASL to assess medullary perfusion.^11^, ^12^, ^17^ The ASL sequence used here was optimized for measurement of cortical perfusion, and importantly, the medulla has comparatively low flow and therefore lower signal and relative precision. With some variation between species, there are differences in the autoregulation of medullary perfusion compared to total and cortical blood flows.^30^, ^31^, ^32^ In rats, medullary autoregulation is reduced during normovolemia^32^ compared with a fluid‐depleted state. Also, the influence of renal sympathetic nerve activity (RSNA) and circulating factors such as angiotensin II and vasopressin affects the cortical and medullary perfusion differently. During normal physiological conditions, RSNA is an external regulator of mainly total renal and cortical blood flow with only modest effects on medullary perfusion.^31^ Angiotensin II increases medullary blood flow during certain conditions^33^ whereas vasopressin mostly reduces medullary blood flow.^30^ A possible explanation for this finding may be a temporary imbalance in the interplay between myogenic autoregulation, the effect of RSNA, and the distributive effects of Angiotensin II and vasopressin. Although we cannot present a probable mechanism for this finding that can explain the phenomenon in the NO AKI group solely, we note that a paradoxical decrease in renal medullary perfusion has been noted during a rapid increase in blood pressure during experimental conditions.^34^ However, the limitations in the study design further elaborated below should also be considered before drawing further conclusions.

In a wider context, fluid bolus administration is a common intervention in the management of ICU patients with limited evidence to guide its proper use. Elevated or increasing P‐Creatinine, as well as mild oliguria, are still used as signs of hypovolemia^35^ where fluid bolus administration is suggested and the expected beneficial effect is mediated by changing renal perfusion. Nevertheless, clinicians' expectations of hemodynamic and renal effects of fluid bolus administration often differ from actual effects.^36^ During the completion of this study, a restrictive approach to fluid management has been recommended also in COVID‐19^27^ based on previous findings in non‐COVID‐19 ARDS.^8^ Furthermore, a previous study has demonstrated that in non‐COVID‐19 ARDS patients, an increase in cardiac index after fluid bolus administration did not correlate to a change in urine output.^37^ Recent clinical trials focusing on the initial management of sepsis have not been able to demonstrate a beneficial effect on AKI development with liberal fluid protocols,^38^ and beyond initial resuscitation, a recent trial in sepsis patients concluded that limiting fluid boluses to patients with more severe and non‐renal triggers did not result in an increase in mortality or the occurrence of severe AKI.^39^ Also, in ICU patients, a fluid restrictive regime may reduce the use of CRRT when AKI is present.^40^ The lack of an increase in renal perfusion seen by plasma expansion in the patients included in our study may further the understanding of the apparent lack of clinical benefit in the studies referred to above.

A limitation of the present study is that severely hypovolemic patients with a resulting circulatory instability were not included in the study. As such, conclusions cannot be made about the effects of plasma expansion on renal perfusion during initial resuscitation and stabilization. This is a common problem in studies where renal blood flow estimation is not readily available bedside. Our study did not include assessment by echocardiography or direct measurement of cardiac output, and the effect on cardiac output by plasma expansion may not have been increased despite MAP elevation.^37^ Also, an increase in central venous pressure by plasma expansion may attenuate the increase in renal perfusion pressure. Still, improvement of hemodynamic variables in general (reduced HR and increased MAP) is a common end point in clinical practice.^36^ We acknowledge that the before–after design is susceptible to bias, including regression to the mean bias.

In conclusion, renal perfusion was not increased shortly after plasma expansion by a standardized fluid bolus of 7.5 mL/kg Ringer's Acetate in normotensive critically ill COVID‐19 patients with AKI or without AKI, respectively.

## AUTHOR CONTRIBUTIONS

TL, PE, SF, JW, FP, ML, PL, and RF contributed to the conception and design of the study. TL, RF, SBA, ML, and MH acquired physiological data and managed patient participation and safety. TL collected clinical patient data. SF provided the imaging protocol. PE, JW, and PL performed imaging acquisition. EC and SF performed imaging analyses. TL, PE, EC, SF, MH, and RF performed data analysis and curation. TL made the formal analysis. The first draft of the manuscript was written by TL. TL prepared images. All authors commented on the manuscript.

## FUNDING INFORMATION

The study was supported with funding from local grants from Uppsala University Hospital, from the NoRCORP (Nottingham Recovery from COVID19 Research Platform), Medical Research Council (grant number MR/V037005/1), the Ernfors Family Foundation, the Selanders Foundation, Region Uppsala, the Swedish Research Council (2014‐02569 and 2014‐07606), The Swedish Kidney Foundation (Njurfonden) (F2020‐0054), SciLifeLab/Knut and Alice Wallenberg national COVID‐19 research program (M.H.: KAW 2020.0182, KAW 2020.0241), the Swedish Heart‐Lung Foundation (M.H.: 20210089, 20190639, 2019063), and the Swedish Society of Medicine (M.H.: SLS‐938101). Funding bodies had no role in the design of the study, data collection, interpretation, or in the writing of the manuscript.

## CONFLICT OF INTEREST STATEMENT

MH and FP are active in the American Physiological Society. The authors declare that they have no relevant conflicts of interest.

## Supporting information


**TABLE S1:** A prospective case control study investigating the effect on renal perfusion by plasma expansion by standardized balanced crystalloid bolus of 7.5 mL/kg in patients with critical COVID‐19 and ARDS divided into two groups depending on AKI status. Renal perfusion was measured using multiparametric MRI using phase contrast and ASL Cortex and Medulla. Additional results are presented by group and in relation to plasma expansion status below. Data are presented as estimated marginal means with 95% confidence intervals derived from the mixed linear model used in the main manuscript in which hypertension and diabetes as dichotomous variables were added to the model as a post hoc sensitivity analysis.

## Data Availability

The data are not publicly available due to privacy or ethical restrictions. Data are however available from the authors upon reasonable request with restrictions as outlined above.
