# Supplementary material for: Plasma expansion and renal perfusion in critical COVID‐19 with AKI: A prospective case control study
Source: Acta Anaesthesiol Scand. 2025 Feb 23;69(3):e70004. doi: 10.1111/aas.70004 (PMC11848233; doi:10.1111/aas.70004)
Supplement: Supplementary file 1 — TABLE S1: A prospective case control study investigating the effect on renal perfusion by plasma expansion by standardized balanced crystalloid bolus of 7.5 mL/kg in patients with critical COVID‐19 and ARDS divided into two groups depending on AKI status. Renal perfusion was measured using multiparametric MRI using phase contrast and ASL Cortex and Medulla. Additional results are presented by group and in relation to plasma expansion status below. Data are presented as estimated marginal means with 95% confidence intervals derived from the mixed linear model used in the main manuscript in which hypertension and diabetes as dichotomous variables were added to the model as a post hoc sensitivity analysis. [file AAS-69-0-s001.pdf]

Supplemenatal table

Manuscript ” Plasma expansion and renal perfusion in critical COVID-19 with AKI: A prospective case control study”

A prospective case control study investigating the effect on renal perfusions by plasma expansion by standardized balanced crystalloid bolus of 7.5ml/kg in patients with critical COVID-19 and ARDS divided in two groups depending on AKI status. Renal perfusions were measured using multiparametric MRI using phase contrast and ASL Cortex and Medulla. Additional results are presented by group and in relation to plasma expansion status below. Data are presented as estimated marginal means with 95% confidence intervals derived from the mixed linear model used in the main manuscript in which hypertension and diabetes as dichotomous variables were added to the model as a *post hoc* sensitivity analysis.

Group	AKI, N=9		NO AKI, N=8	
Plasma expansion status	Before	After	Before	After
Total renal blood flows, ml/min/kidney	345 (232-457)	358 (246-470)	418 (308-529)	413 (302-523)
Cortical perfusion, ml/min/100g	98 (69-127)	101 (72-131)	146(118-175)	129(100-158)
Medullary perfusion†, ml/min/100g	32 (22-47)	34 (23-49)	57 (39-80)	34 (24-49)**

** indicates p-value < 0.01in pairwise comparison within group after Tukey’s adjustment

† indicates p-value < 0.05 of before-after effect of plasma expansion and interaction effect in ANOVA
